# Efficacy of an automatic electric toothbrush with nylon bristles in dental plaque removal: a cross-over randomized controlled trial

**DOI:** 10.1007/s00784-024-05601-w

**Published:** 2024-03-14

**Authors:** Maria Denisa Statie, Irene Lomonaco, Michele Nieri, Veronica Giuntini, Debora Franceschi, Lorenzo Franchi

**Affiliations:** 1 Graduate Orthodontic Program, Department of Experimental and Clinical Medicine, Università degli Studi di Firenze, Florence, Italy; 2 Department of Experimental and Clinical Medicine, Università degli Studi di Firenze, Via del Ponte di Mezzo 46-48, Florence, 50127 Italy

**Keywords:** Randomized controlled trial, Dental plaque, Powered toothbrush, Mtoothbrush, Oral hygiene

## Abstract

**Objectives:**

The objective of this single-use, five-treatment, five-period, cross-over randomized controlled trial (RCT) was to compare the efficacy in dental plaque removal of a new Y-shaped automatic electric toothbrush (Y-brush) compared to a U-shaped automatic electric toothbrush (U-brush), a manual toothbrushing procedure (for 45 and 120 s), and no brushing (negative control).

**Materials and methods:**

Eligible participants were volunteer students randomized to the treatments in the five periods of the study. The primary outcome measure was the reduction in full-mouth plaque score (FMPS) after brushing while the secondary outcome variable was a visual analogic scale (VAS) on subjective clean mouth sensation. Mixed models were performed for difference in FMPS and VAS.

**Results:**

After brushing procedures, manual toothbrushing (120 s) showed a statistically significant reduction in FMPS than Y-brush (difference 36.9; 95%CI 29.6 to 44.1, *p* < 0.0001), U-brush (difference 42.3; 95%CI 35.1 to 49.6, *p* < 0.0001), manual brushing (45 s) (difference 13.8; 95%CI 6.5 to 21.1, *p* < 0.0001), and No brushing (difference 46.6; 95%CI 39.3 to 53.9, *p* < 0.0001). Y-brush was significantly more effective than No brushing (difference 9.8; 95%CI 2.5 to 17.0, *p* = 0.0030), while there was no significant difference compared to U- brush. Similar results were obtained for the differences in the Clean Mouth VAS.

**Conclusions:**

Y-brush was significantly more effective than no brushing (negative control) in removing dental plaque. When compared to manual toothbrushing for both 45 and 120 s, however, Y-brush was less effective in dental plaque removal.

**Clinical relevance:**

Modified design of automatic toothbrushing devices could improve plaque reduction, especially in patients with intellectual disabilities or motor difficulties.

## Introduction

Dental plaque biofilm is a microbial community responsible for the most common oral diseases such as dental caries and periodontal disease [1]. The build-up of plaque deposits on tooth surfaces impairs oral health and increases the incidence of disease within the oral cavity [2]. The toothbrush is an important tool in daily oral care that facilitates the removal of plaque deposits and the prevention of their damaging effects [3].

The toothbrush has acquired an important value in personal care that makes it an indispensable accessory in everyday life for many people. Indeed, in recent years, the production of both manual and electric toothbrushes increased exponentially to satisfy the population needs and habits [4]. Consequently, this prompted more interest in investigating the efficacy of the various types of toothbrushes.

A randomized clinical trial (RCT) that evaluated the effectiveness of 11 different types of manual toothbrushes in removing plaque deposits, revealed no statistically significant differences between them [4]. However, some systematic reviews showed better efficacy of the electric toothbrush compared to the manual toothbrush in removing plaque and improving gingivitis [5, 6].

Despite the increasing popularity of the U-shaped automatic electric toothbrush (U-brush) with silicone bristles and simultaneous action on both arches, a randomized trial showed that the U-brush was not effective in removing dental plaque [7].

However, a new Y-shaped automatic electric toothbrush (Y-brush) with nylon bristles and with action on each separate arch, has recently been proposed in a pilot RCT [8].

The objective of the present cross-over RCT was to compare the efficacy in dental plaque removal of Y-brush compared to the U-brush, a manual toothbrushing procedure (for 45 and 120 s), and no brushing.

## Materials and methods

The experiment design followed the Consolidated Standards of Reporting Trials (CONSORT) statement [9].

### Ethics and consent to participate

The principles outlined in the Declaration of Helsinki on clinical research involving human subjects were adhered to. The study was approved on July 17th, 2022, by the ethical committee (Comitato Etico Regione Toscana Area Vasta Centro, approval number 22019_spe). Written informed consent was obtained from all study participants.

### Protocol registration

The study was registered on ClinicalTrials.gov with registration number NCT05594134 on October 26th, 2022 (https://clinicaltrials.gov/ct2/show/study/NCT05594134).

#### Trial design

This was a single-use, five-treatment, five-period (visit), cross-over, mono-centered, examiner-blind randomized controlled trial with treatment sequences balanced for carryover effects.

There were five treatments per subject assigned in a randomized order:

1. No brushing (negative control) (No brushing group).
2. Y-shaped automatic electric toothbrush (Y-brush group).
3. Manual toothbrush 45 s (Manual 45 group).
4. Manual toothbrush 120 s (Manual 120 group).
5. U-shaped automatic electric toothbrush (silicon bristles) (U-brush group).

#### Participants

The inclusion criteria of participants were volunteer students of the 6th year of the School of Dentistry and to the residents of the Graduate Orthodontic Program and of the Graduate Oral Surgery Program of the University of Florence. The participants had to be aged between 18 and 30 years with presence of at least 20 teeth, no fixed orthodontic appliance, and full-mouth plaque score (FMPS) at each visit above 40% [10].

Exclusion criteria were participants with manual disabilities to perform normal oral hygiene maneuvers and participants allergic to silicone and nylon.

All students involved in the study had to have been already evaluated by the investigators in their curriculum. The study took place at the University of Florence during the period between November and December 2022.

A week before the start of the study all participants received all the toothbrushes investigated in this study. Additionally, all students participated in a demonstration session on the use of each one of the toothbrushes.

#### Interventions

Participants were instructed by one of the investigators (V.G.) to refrain from all oral hygiene procedures, from rinsing with mouthwash, and from chewing gum for approximately 12 h prior to their appointment time. Participants had to bring always all toothbrushes at each visit.

At the first visit, participants who had given signed informed consent, and who were eligible in terms of the inclusion and exclusion criteria, entered the study. At each appointment participants were tested for the amount of bacterial plaque on their teeth before and after brushing by one of the investigators (D.F.). A solution (Mira-2-Ton, Hager Werken, Duisburg, Germany) was applied to the teeth of the participants with a cotton pellet to disclose their dental plaque. The examiner (D.F.) then performed a baseline plaque examination with a magnifying system (EyeMag Pro S 4.5X, Zeiss, Jena, Germany) using the full-mouth plaque score (FMPS) on 6 sites per tooth [10]. Afterward, the examiner left the room while another operator (V.G.) opened an opaque and sealed envelope containing the random assigned procedure. Each subject was instructed by one of the investigators (V.G.) to brush her/his teeth with the randomized assigned toothbrush, without toothpaste, under supervision, and with the aid of a mirror according to the instructions.

After that the subject had brushed her/his teeth, the examiner (D.F.) went back into the room and performed a second plaque examination. The same procedure was followed for each of the visits in turn, which were separated by an interval of at least 7 days. At each visit, participants were assigned to procedures according to their treatment sequence. Participants were assessed at each visit for their eligibility to continue in the study (FMPS above 40%).

**Fig. 1:**
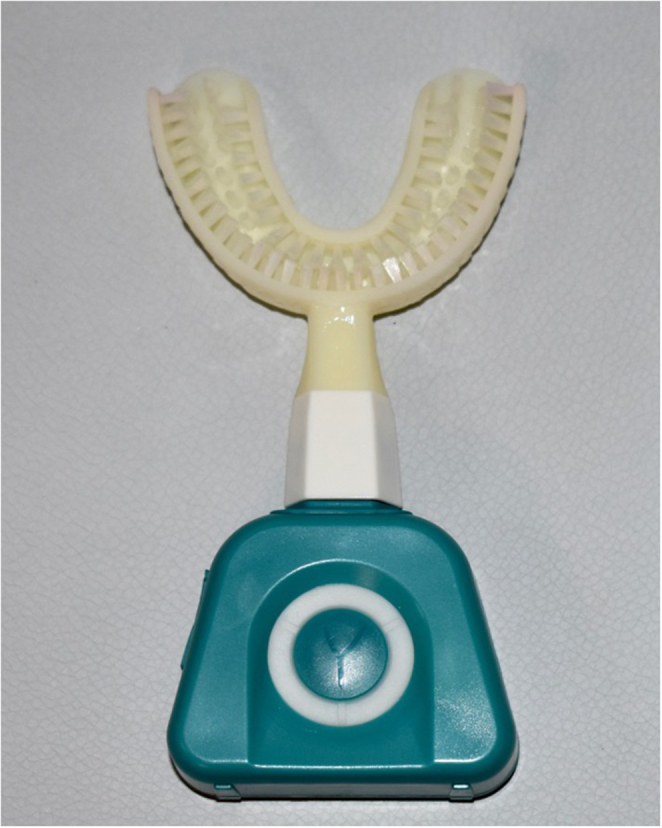
The Y-shaped automatic electric toothbrush with nylon bristles

**Fig. 2:**
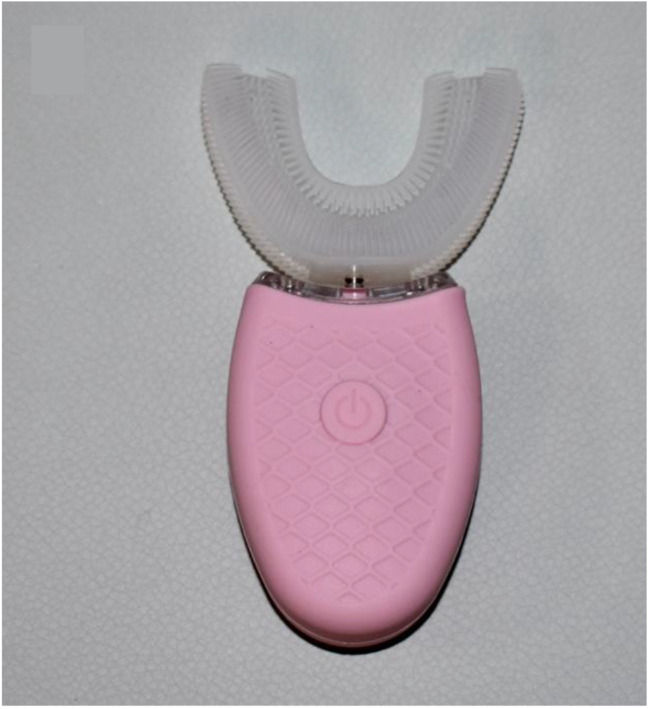
The U-shaped automatic electric toothbrush with silicon bristles

There were five treatments per subject assigned in a randomized order:

1. No brushing (negative control) (No brushing group). Participants were asked not to brush their teeth and wait two minutes at rest.
2. Y-shaped automatic electric toothbrush (Y-Brush, Caluire-et-Cuire, Lyon, France). Participants were asked to brush their teeth for 10 s per arch with the Y-brush (nylon bristles) without toothpaste. Participants had to press the “Y” button to power on the brush and then to press the button twice to set the duration of the cycle of brushing to 10 s (Fig. 1).

3. Manual toothbrush (Manual 45, Oral B Cross Action, Procter & Gamble, Cincinnati, OH, USA). Participants were asked to brush their teeth with manual toothbrush for 45 s without toothpaste.
4. Manual toothbrush (Manual 120, Oral B Cross Action). Participants were asked to brush their teeth with manual toothbrush for 120 s without toothpaste.
5. U-shaped automatic electric toothbrush with silicon bristles (U-Shaped Toothbrush, YUYTEnhmcsibu6t959-11, China). Participants were asked to brush their teeth for 10 s with the U-shaped automatic electric toothbrush (silicon bristles) without toothpaste (Fig. 2).

#### Outcomes

The primary outcome measure was the difference in FMPS between before and after brushing. The examiner (D.F.) performed the plaque assessment using a magnifying system (EyeMag Pro S 4.5X, Zeiss, Jena, Germany) and registered the presence or absence of plaque on 6 sites per tooth. The examiner was blinded to the allocated treatment. The FMPS was expressed as a percentage (number of sites with plaque on the total of examined sites) [10] to evaluate the effect of brushing on all teeth concurrently. The operator had been assessed before the study for an intra-rater reproducibility by measuring 738 sites two times after a washed-out period of two hours. The kappa statistic was 0.95 (95% CI from 0.93 to 0.98) [7].

The secondary outcome variable was a visual analogic scale (VAS) on subjective clean mouth sensation of the participants. The minimum value (0) was no clean mouth sensation, and the maximum value (10) was best sensation of clean mouth. This VAS was registered by the participant after each brushing period before the second plaque evaluation.

#### Sample size

Considering a clinically relevant difference in FMPS of 15, a standard deviation of 12.90 [2], a two-tailed statistical significance threshold of α = 0.005 (Bonferroni correction), and a power of 80%, a sample size of 25 participants was necessary given an anticipated drop-out rate of 10%.

#### Randomization

The randomization list was computer generated taking into account the fact that each subject performed all 5 treatments and that the treatments were balanced within the 5 periods (visits).

The allocation sequence was concealed from the researcher (M.N.) enrolling and assessing participants in sequentially numbered, opaque, and sealed envelopes. The number on the envelope identified the patient and the visit. The envelopes were opened only when the treatment was assigned by one operator (V.G.) after that the examiner (D.F.) had left the room.

#### Blinding

While the operator and patients were aware of the allocation arm, the outcome assessor was kept blinded to the allocation period.

#### Statistical methods

Descriptive statistics were performed using mean and standard deviation. Analysis of covariance (ANCOVA) was performed. Tukey method for pairwise comparison was carried out.

A mixed model was performed for the difference in FMPS. In the model, the random effect was represented by the subject and the fixed effects were represented by the type of intervention (No brushing, Y-brush, Manual 45, Manual 120, U-Brush), the period (1, 2, 3, 4, 5), and the covariate represented by the FMPS registered before the brushing period. The period was added to the models only if significant. In case of statistical significance of the type of intervention, Tukey’s *post hoc* test was carried out.

A mixed model was implemented also for “clean mouth” sensation assessed on the VAS. In the model, the random effect was represented by the subject and the fixed effects were represented by the type of intervention (No brushing, Y-brush, Manual 45, Manual 120, U-Brush) and the period (1, 2, 3, 4, 5). The period was added to the models only if significant. In case of statistical significance of the type of intervention, Tukey’s *post hoc* test was performed.

Estimates for the treatment effect, p-values, and 95% confidence intervals were provided. The statistical software was JMP (version 13, SAS Institute Inc., Cary, NC, USA).

## Results

**Fig. 3:**
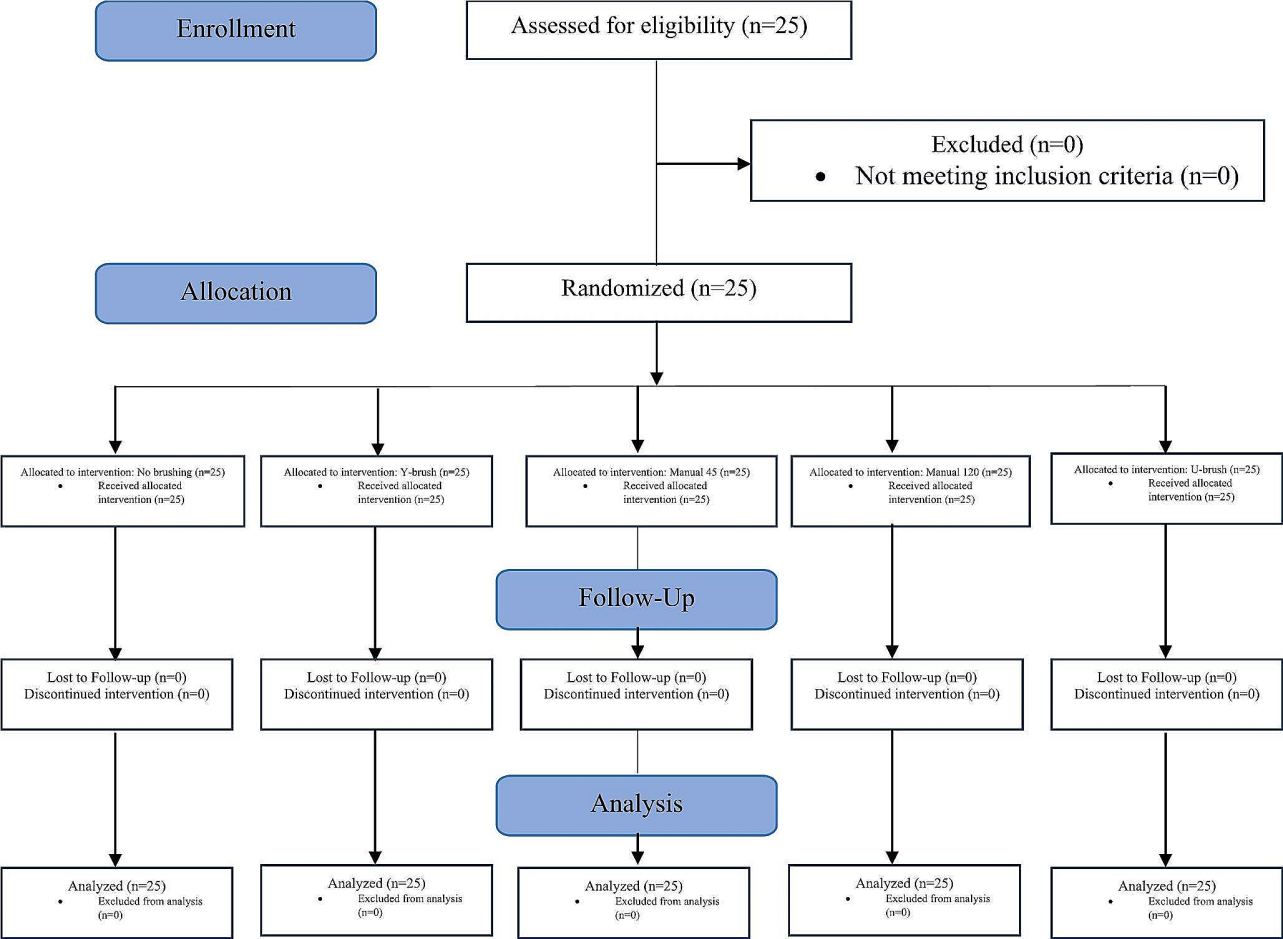
CONSORT flow diagram

This crossover randomized controlled trial was carried out upon 25 volunteer students who were randomized to the treatments in the five periods of the study, comparing 5 different treatments for plaque removal (Fig. 3). Participants were recruited from September to October 2022 and the study was completed by December 2022. There were no dropouts and there were no deviations from the planned protocol. Thirteen females (52%) and 12 males (48%) took part to the study. The mean age of the participants was 25.6 years (SD 1.5; min 23 years; max 29 years). Twelve participants (48%) used habitually manual conventional toothbrushes and thirteen participants (52%) used habitually powered conventional toothbrushes. Four participants (16%) were smokers, smoking up to 5 cigarettes per day. The number of teeth for each patient was on average 29.6 (SD 1.9; min 26; max 32). The FMPS before and after the brushing period, the FMPS difference, and the clean mouth VAS for each treatment are reported in Table 1. There were no differences between treatments in FMPS before the brushing period. The mean difference in FMPS based on the period (visit) is reported in Table 2. In the ANCOVA for FMPS reduction (difference between FMPS before and after brushing), the treatment was significant (*P* < 0.0001) and also the period was significant (*P* = 0.0050). The difference in FMPS reduction between treatments using Tukey’s *post hoc* method for pairwise comparison is reported in Table 3.

**Table 1:** Descriptive statistics. Mean full-mouth plaque score (FMPS) before (T0) and after (T1) the brushing period, FMPS reduction and clean mouth visual analogic scale (VAS) for each treatment. The standard deviation is between parentheses

	No brushing^a^ *N* = 25	Y-brush^b^ *N* = 25	Manual 45^c^ *N* = 25	Manual 120^d^ *N* = 25	U-brush^e^ *N* = 25
FMPS T0	71.6 (12.8)	69.5 (13.9)	72.1 (12.9)	72.0 (13.6)	71.4 (13.6)
FMPS T1	65.4 (14.9)	54.1 (14.7)	32.9 (14.7)	19.0 (11.5)	60.9 (14.5)
FMPS reduction	6.2 (5.2)	15.4 (9.0)	39.2 (14.4)	53.0 (14.4)	10.5 (6.7)
Clean mouth VAS	1.8 (1.8)	3.7 (1.6)	5.6 (1.5)	7.6 (1.3)	3.2 (1.6)

^a^No brushing: negative control

^b^Y-brush: Y-shaped automatic electric toothbrush

^c^Manual 45: manual toothbrush 45 s

^d^Manual 120: Manual toothbrush 120 s

^e^U-brush: U-shaped automatic electric toothbrush

**Table 2:** FMPS reduction based on period (visit). The standard deviation is between parentheses

	First visit	Second visit	Third visit	Fourth visit	Fifth visit
FMPS reduction	19.1 (15.5)	26.0 (25.3)	28.3 (25.2)	25.3 (19.0)	25.6 (18.7)

**Table 3:** Difference in FMPS reduction between treatments using Tukey method for pairwise comparison

Treatment 1	Treatment 2	Difference in FMPS reduction	95%CI	P-value
Manual 120^a^	No brushing^b^	46.6	39.3; 53.9	< 0.0001
Manual 120	U-brush^c^	42.3	35.1; 49.6	< 0.0001
Manual 120	Y-brush^d^	36.9	29.6; 44.1	< 0.0001
Manual 45^e^	No brushing	32.8	25.6; 40.1	< 0.0001
Manual 45	U-brush	28.5	21.3; 35.8	< 0.0001
Manual 45	Y-brush	23.1	15.8; 30.4	< 0.0001
Manual 120	Manual 45	13.8	6.5; 21.1	< 0.0001
Y-brush	No brushing	9.8	2.5; 17.0	0.0030
Y-brush	U-brush	5.5	-1.8; 12.7	0.2347
U-brush	No brushing	4.3	-3.0; 11.6	0.4736

^a^Manual 120: manual toothbrush 120 s

^b^No brushing: negative control

^c^U-brush: U-shaped automatic electric toothbrush

^d^Y-brush: Y-shaped automatic electric toothbrush

^e^Manual 45: manual toothbrush 45 s

The differences between treatments in FMPS reduction between before and after brushing resulted statistically significant between the manual 120 and No brushing (difference 46.6; 95% CI from 39.3 to 53.9, *p* < 0.0001) favoring the manual 120, between the manual 120 and U-brush (difference 42.3; 95% CI from 35.1 to 49.6; *P* < 0.0001) favoring the manual 120, between the manual 120 and Y-brush (difference 36.9; 95% CI from 29.6 to 44.1; *P* < 0.0001) favoring the manual 120, between the manual 45 and No brushing (difference 32.8; 95% CI from 25.6 to 40.1; *P* < 0.0001) favoring the manual 45, between the manual 45 and U-brush (difference 28.5; 95% CI from 21.3 to 35.8; *P* < 0.0001) favoring the manual 45, between the manual 45 and Y-brush (difference 23.1; 95% CI from 15.8 to 30.4; *P* < 0.0001) favoring the manual 45, and between the manual 120 and manual 45 (difference 13.8; 95% CI from 6.5 to 21.1; *P* < 0.0001) favoring the manual 120. The difference between the Y-brush and No brushing was significant (difference 9.8; 95% CI from 2.5 to 17.0; *P* = 0.0030) favoring the Y-brush. On the contrary, the difference between the Y-brush and U-brush was not significant (difference 5.5; 95% CI from − 1.8 to 12.7; *P* = 0.2347) favoring the Y-brush. Another not significant difference was found between the U-brush and No brushing (difference 4.3; 95% CI from − 3.0 to 11.6; 0.4736) favoring the U-brush (Table 3).

During the first visit the subjects removed less plaque. During the following four visits the participants removed more plaque than the first visit regardless of the treatment performed (Table 2).

The difference between treatments in Clean Mouth VAS was significant (*P* < 0.0001) while the period was not significant. The difference in Clean Mouth VAS using Tukey’s *post hoc* method for pairwise comparison is reported in Table 4.

**Table 4:** Difference in the Clean Mouth VAS (ANOVA) using Tukey method for pairwise comparison

Treatment 1	Treatment 2	Difference in Clean Mouth VAS	95%CI	P-value
Manual 120^a^	No brushing^b^	5.8	4.8; 6.9	< 0.0001
Manual 120	U-brush^c^	4.5	3.4; 5.5	< 0.0001
Manual 120	Y-brush^d^	3.9	2.9; 5.0	< 0.0001
Manual 45^e^	No brushing	3.8	2.7; 4.9	< 0.0001
Manual 45	U-brush	2.4	1.4; 3.5	< 0.0001
Manual 120	Manual 45	2.0	1.0; 3.1	< 0.0001
Y-brush	No brushing	1.9	0.9; 3.0	< 0.0001
Manual 45	Y-brush	1.9	0.8; 2.9	< 0.0001
U-brush	No brushing	1.4	0.3; 2.4	0.0054
Y-brush	U-brush	0.6	-0.5; 1.6	0.5909

^a^Manual 120: manual toothbrush 120 s

^b^No brushing: negative control

^c^U-brush: U-shaped automatic electric toothbrush

^d^Y-brush: Y-shaped automatic electric toothbrush

^e^Manual 45: manual toothbrush 45 s

The differences in Clean Mouth VAS were statistically significant comparing Manual 120 with No brushing, U-brush, Y-brush, and Manual 45. The differences were statistically significant comparing Manual 45 with No brushing, U-brush, and Y-brush. The difference was statistically significant also comparing Y-brush with No brushing. On the contrary no statistically significant differences were detected between U-brush vs. No brushing and Y-brush vs. U-brush (Table 4).

## Discussion

The present cross-over RCT aimed to compare the efficacy in dental plaque removal of a Y-shaped automatic electric toothbrush (Y-brush) compared to a manual toothbrushing procedure (for 45 or 120 s), a U-shaped automatic electric toothbrush (U-brush), and No brushing. In particular, this study was made to test the efficacy of a new Y-shaped automatic electric toothbrush with nylon bristles with a fully automatic action on each arch that has been proposed recently.

Our study showed that in general a manual toothbrushing procedure was more effective in dental plaque reduction when compared to either Y-brush, U-brush or No brushing.

The duration of toothbrushing with a manual toothbrush was set at 45 or at 120 s depending on treatment. The difference between manual toothbrushing for 45 and 120 s was statistically significant, showing a deeper cleansing action during the procedure that required more brushing time. The recommended time for toothbrushing with a Y-brush and with a U-brush was shorter than manual toothbrushing (10 s per arch for the Y brush and 10 s in both arches, for the U-Brush).

The results of this cross-over RCT showed that Y-brush was significantly more effective than No brushing in removing dental plaque. When compared to manual toothbrushing for both 45 and 120 s, however, Y-brush was less effective in dental plaque removal. This outcome could be probably related to the fact that the nylon bristles were too short and did not reach the dental or gingival surfaces effectively, or also to the fact that a greater amount of brushing time with this new device could be needed. Additionally, the mouthpiece is fixed in shape and size and, therefore, it may not fit the individual dental arch shape and size. Nevertheless, in this study the size of the dental arch of the participants was not measured.

The results of the present study agree with a recent pilot RCT on the efficacy of Y-brush compared to manual brushing by Keller et al. [8]. They found that full-mouth plaque reduction was higher with manual toothbrushing than Y-brush used for 5 s. For evaluating the effect of longer brushing with the Y Brush, Keller et al. [8] increased the brushing time from 5 to 15 s per arch. Ten volunteers were willing to participate in this second part of the study (non-blinded, nonrandomized). When the brushing time of with Y-Brush was increased to 15 s per arch, the reduction in whole mouth plaque scores was significantly higher than for the 5-s brushing mode and not significantly different compared to manual toothbrushing.

The results of U-Brush in reduction of FMPS were very similar to those reported in a previous study [7] that showed that there was no significant difference even when compared to No brushing. In a recent study investigating an auto-cleaning device with silicon bristles (Amabrush®, Vienna, Austria), very similar to the U-Brush, Schnabl et al. [11] found that none of the subjects reached an equal or higher plaque reduction with Amabrush compared to manual toothbrushing. It should be noted that Amabrush is no longer available. However, other similar U-brushes are available in the market.

As for the subjective clean mouth sensation, the results of the present study were similar to FMPS reduction with best scores for the manual 120 group when compared to Y-brush, U-brush and No brushing. The manual 45 group showed a statistically significant difference when compared to U-brush and No brushing, while it showed no significant difference when compared to the Y-brush group.

Despite showing no statistically significant differences between each other, Y-brush and U-brush resulted to have best scores in subjective clean mouth sensation when compared to No brushing.

During the first visit the participants removed less plaque compared to the following four visits regardless of the treatment performed. The reason for this result is unclear. Probably the participants were less effective in the first visit because they were anxious or because they developed a so called “negative Hawthorne effect” [12].

A limitation of this study was that the five tested procedures were performed “one-shot”. No long-term effects, therefore, could be assessed. Moreover, we could not report any adverse effect that could be related to a more prolonged use of the Y-brush. Other variables, like gingivitis, were not assessed in the present study. Another limitation was that the present study was not performed on patients but rather on undergraduate students of the School of Dentistry and on postgraduate students. This aspect could limit the generalizability of the results also because about half of the participants used habitually manual conventional toothbrushes. In this study brushing was carried out without toothpaste to compare the mechanical action of the toothbrushes in plaque removal. The use of a toothpaste could have modified the results of this study.

In a Cochrane systematic review, Waldron et al. [13] found that people with an intellectual disability showed a greater severity and a higher prevalence of periodontal disease than the general population. Moreover, their oral health got worse at a faster rate as they moved into adulthood. Therefore, automized toothbrushing devices like the Y-brush or U-brush could be potentially useful especially in patients with intellectual disability or in patients with motor difficulties. Manufacturers, however, should try to modify the design of these devices to improve their efficacy in dental plaque removal.

## Conclusions

Y-brush was significantly more effective than no brushing (negative control) in removing dental plaque. When compared to manual toothbrushing for both 45 and 120 s, however, Y-brush was less effective in dental plaque removal. Y-brush could be taken into account in patients with intellectual disability or in patients with motor difficulties.

## Data Availability

The data will be available on request.
